# High expression of estrogen receptor alpha and aromatase in glial tumor cells is associated with gender-independent survival benefits in glioblastoma patients

**DOI:** 10.1007/s11060-020-03467-y

**Published:** 2020-04-02

**Authors:** Lisa Stefanie Hönikl, Friederike Lämmer, Jens Gempt, Bernhard Meyer, Jürgen Schlegel, Claire Delbridge

**Affiliations:** 1 Department of Neuropathology, Klinikum rechts der Isar, School of Medicine, Technical University of Munich, Munich, Germany; 2 Department of Neurosurgery, Klinikum rechts der Isar, School of Medicine, Technical University of Munich, Munich, Germany

**Keywords:** Glioblastoma multiforme, Estrogen receptor alpha, Aromatase, 17β-estradiol, Survival, TMZ, Combined therapy in vitro

## Abstract

**Introduction:**

Glioblastoma multiforme (GBM) is a highly malignant glial tumor, affecting men more often than women. The reason for this gender-specific predominance remains unclear, raising the question whether these effects are subject to hormonal control. The purpose of this study was to examine the expression of estrogen receptor alpha (ERα) and aromatase in human GBM tissue samples in relation to patient survival and furthermore to investigate the effect of standard chemotherapy in combination with estradiol treatment on glioblastoma tumor cell lines in vitro.

**Methods:**

60 tissue samples (31 male, 29 female) of GBM patients were analysed with immunohistochemistry for ERα and aromatase for survival analyses. The cell lines LN18 and LN229 were treated with 17β-estradiol (E2) in different dosing regimens and the cell viability was measured with MTT assay. After estradiol pre-treatment Temozolomide was added and tested again.

**Results:**

High expression of ERα and aromatase in the GBM tissue samples was associated with significantly longer survival times of GBM patients, regardless of gender and body-mass-index. The treatment with high concentrations of estradiol resulted in lower tumor cell viability, compared to control. The cells significantly showed a stronger sensitivity against Temozolomid (TMZ) after estradiol pre-treatment.

**Conclusion:**

ERα-expression of glial tumour cells seems to play an important prognostic role as a biomarker in GBM, as well as the expression of the enzyme Aromatase. The combined treatment of GBM with standard chemotherapy and estradiol may be beneficial to patient’s survival.

## Introduction

Glioblastoma multiforme (GBM, WHO grade IV) is the most common and most malignant primary brain tumor in adults [1]. Despite maximal standard therapy [2] the prognosis is still poor and leads to a median overall survival of 15 months [3]. The incidence of GBM in the USA was 3.2/100,000 person years in 2015 and men are affected 1.6 times more often than women [4]. The reason for this gender-specific predominance still is unclear.

Many neurological diseases like traumatic brain injury [5], stroke [6] and chronic progressive course [7] also show gender differences, which are most likely attributed to the neurobiology of reactive astrocytes and the expression of estrogen, estrogen receptors (ER) and aromatase. In traumatic brain injury female rats showed smaller tissue damages compared to male and ovariectomized female rats [8]. Female homozygous aromatase knock-out mice showed larger ischemic damage after reversible cerebral artery occlusion than the wild-type littermates, suggesting that the enzyme plays a key role in neuroprotective functions [9]. The neuroprotective functions of sexual steroid hormones are mainly mediated through ERs in astrocytes [10]. When GBM cells were transplanted into athymic mice [11] or nude rats [12] the tumor growth was higher in male than female animals, indicating that sexual hormones have a decisive influence on tumor proliferation.

Aromatase converts testosterone to estradiol as last step of estrogen biosynthesis. The enzyme, located in the endoplasmic reticulum, is expressed in the gonads, glial cells, neurons and the adipose tissue [13]. The main site of synthesis under physiological conditions in the brain are neurons, but there is also notable expression in glial cells [14]. Under pathological conditions the production shifts to glial cells, mainly astrocytes [15, 16].

Estradiol is a steroid hormone, synthesized from cholesterol in different organs: the gonads, the adrenals, the placenta and the brain. In the brain estrogen levels depend on the synthesis of estrogens by neurons and glial cells de novo but also on the absorption of blood derived estrogens [17, 18]. Estrogen, as a steroid hormone, acts directly by binding nuclear ERs and initiates gene expression under physiological and pathological conditions [15]. There are two isoforms of estrogen receptors: α and β. Differences in ER subtypes suggest diverse function and tumor suppressive properties. Both receptors are expressed under physiological conditions and are lost or reduced during tumor development, indicating a potential tumor suppressive function [19]. Studies concerning cerebral ischemia show that neuroprotective effects are mediated by ERα and not ERβ, because the neuroprotection through estrogen is dependent on the presence of ERα [20, 21].

The aim of this study was to evaluate the expression of ERα and aromatase in tissue samples of glioblastoma patients and to have a closer look at the reaction of glioblastoma cell lines to a combined therapy with estrogen and Temozolomid (TMZ).

## Materials and methods

### Immunohistochemistry

GBM tissue samples were surgically removed, formalin fixed, paraffin embedded and diagnosed as glioblastoma, IDH-wildtype (WHO grade IV), by two independent neuropathologists at our Department of Neuropathology.

Paraffin embedded tumor tissue was cut and placed on microscope slides. After deparaffinising and boiling in 10 mM citrate buffer for 30 min, the endogenous peroxidase was quenched in 1.5% H_2_O_2_ for 10 min. Avidin/Biotin Blocking Kit (Vector Laboratories Inc.) diluted in blocking buffer (PBS plus 1% BSA, 0.1% Trixon 100, 0.2% Gold Fish Gelatine, 0.02% NA acid, 2.5% normal horse serum) was used for 30 min. After washing in PBS the sections were incubated with an ERα antibody (DCS Innovative Diagnostik-Systeme GmbH, 1:20, clone 6F11) or an aromatase antibody (Sigma-Aldrich Co., 1:150, clone 19A1) diluted in blocking buffer overnight at 4 °C. The slides were washed in PBS and incubated with the secondary antibody for 30 min at room temperature (RT). After washing in PBS and incubation with Vecastan ABC Kit (Vector Laboratories Inc.) for 30 min at RT, ImmPACT DAB (Vector Laboratories Inc.) was used for developing under visual control. Counterstaining was done with Meyer’s Haematoxylin and mounted with Pertex. Placenta and breast tissue were used as positive controls. For negative controls the first antibody was omitted.

The slides were quantitatively assessed (10 fields of vision in 20 fold magnification) and scaled in four staining groups: 1 = 0–10%, 2 = 11–40%, 3 = 41–70%, 4 = 71–100% of immune positive glial tumor cells in the vital tumor spreading regions. The Aromatase immunohistochemistry showed a distinct staining of the cytoplasm of glial tumor cells, which were rated as positive. The used ER antibody stains the alpha subtype. The glial tumor cells showed ERα positive cell nuclei, but also cytoplasmic expression of ERs [15, 22]. For the assessment a tumor cell was rated positive when the cell nuclei alone or combined with the cytoplasm was positively stained.

### Cell culture

GBM cell lines LN229 and LN18 were obtained from the American Type Culture Collection and cultivated in 75 cm^2^ flasks in a humidified 5% CO_2_/95% air incubator in high glucose DMEM supplemented with 5% fetal calf serum and 1% l-Glutamine at 37 °C.

### MTT assay

Cell numbers were investigated using MTT assay. Briefly, cells were seeded in 96 well plates in 100 µl medium. After finishing the experiment 10 µl of the MTT labelling reagent (Sigma-Aldrich Co.) was added and incubated for 4 h in a humidified atmosphere. Then the medium was aspirated and 100 µl of the solubilisation solution DMSO was added and incubated for 30 min shaking at RT. After the incubation period a spectrophotometrically absorbance measurement was done at 595 nm (650 nm reference wavelength). As control a triplet of wells per plate was treated under experimental conditions without any cells.

### Western blot

GBM cell lines LN18 and LN229 were analysed for ERα protein. As positive control Gibco®Human Astrocytes (Life Technologies Corp.) were used, which express ERα under physiological conditions [15]. Cells were lysed in RIPA buffer (10 mM HEPES, 150 mM NaCl, 2 mM EDTA, 1% NP 40, 1% Trixon and 10 µl/ml RIPA PMSF, 10 µl/ml RIPA proteinase-phosphatase-inhibitor), transferred to an Eppendorf tube, sonicated for 30 s, incubated on ice for 15 min and centrifuged at 14,000 U/min for 10 min at 4 °C. The resulting supernate was used for the Bradford calculation and the other part was diluted 5:1 in Lämmli-buffer [10% SDS, 125 mM Tris, 10% Glycerol, 0.2% Bromophenol solution, 250 mM DTT (fresh added)], boiled for 5 min and electrophoresed on a 10% SDS-PAGE gel. The protein transfer to the nitrocellulose membrane was done as a gel sandwich inside a tray in a transfer tank filled with transfer buffer at 4 °C for 1 h with 100 V. The membrane was rinsed in TBS with 0.1% Tween 20 (TBST), blocked in 5% bovine serum albumin in TBST for 1 h at RT and incubated with ERα antibody (6F11, Thermo Fisher Scientific Inc., 1:500) at 4 °C overnight. After several rinses in TBST, the membranes got incubated with HRP-linked horse anti-mouse antibody (1:5000 in 5% BSA diluted in TBST) for 45 min at RT. Incubation with the HRP substrate (Merck KGaA) was performed after washing with TBST. After exposure of a film to the HRP wetted membrane the bands were visualized. Vinculin was used for the loading control.

### E2 and TMZ therapy

Cells were seeded into a 96 well plate and treated 24 h later with estradiol (17β-estradiol: E8875, Sigma-Aldrich Co.) in three different dosing regimens: 10 μM, 25 μM and 50 μM diluted as described in the data information. After 48 h of incubation the cell viability was measured with MTT assay. After the estradiol treatment TMZ (Sigma-Aldrich Co.) was added in five different concentrations: 25 µM, 50 µM, 100 µM, 200 µM, 400 µM for 5 days and tested with MTT assay.

### Statistical analysis

Excel 2016 and SPSS statistics 24 were used for all statistical analysis. Survival analysis was plotted on Kaplan–Meier curves according to the REMARK guidelines [23], adjusted to sex and body-mass-index. The Mantel-Cox lag rank test was used to compare survival distributions.

## Results

### GBM cohort analysis of ERα and aromatase

The GBM tissue samples, assessed as described in Materials/Methods and scaled in four groups: 1 = 0–10%, 2 = 11–40%, 3 = 41–70%, 4 = 71–100% of immune positive glial tumor cells. The classification resulted from the fact that many tissue samples showed no or only a small amount of ERα-positive stained tumor cells. Thus a clearer differentiation between less stained tissue samples was possible. For further classifications 0–40% (group 1 and 2) of immune positive stained tumor cells was rated as low expression of the hormones and 41–100% was classified as high expression.

**Fig. 1:**
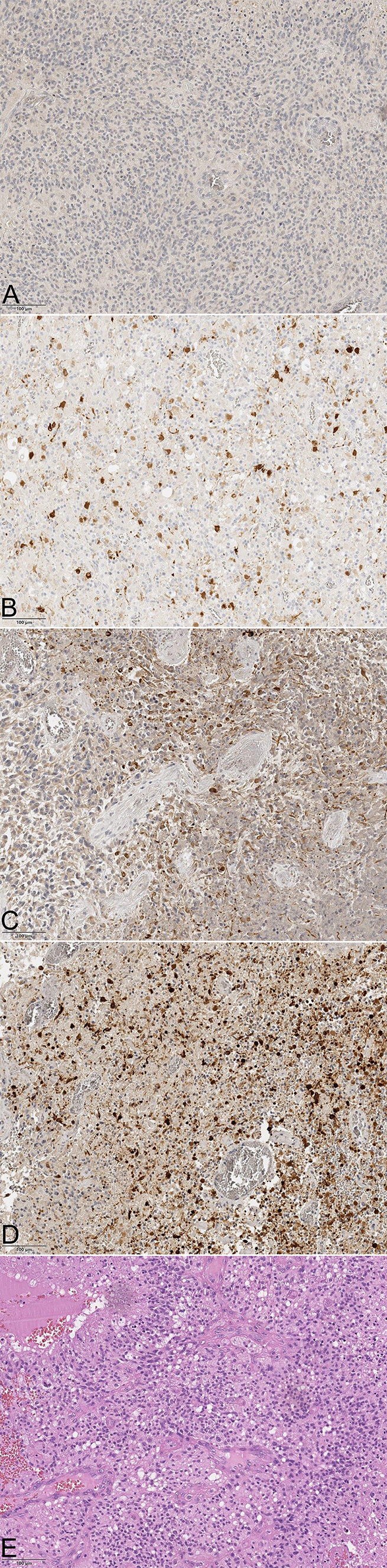
**a** ERα staining of GBM patients in each category (× 20). Picture **a** represents group 1 (0–10%), **b** is group 2 (11–40%), **c** group 3 (41–70%) and **d** is group 4 (71–100%). In picture **e** the hematoxylin eosin stainging is shown

**Fig. 2:**
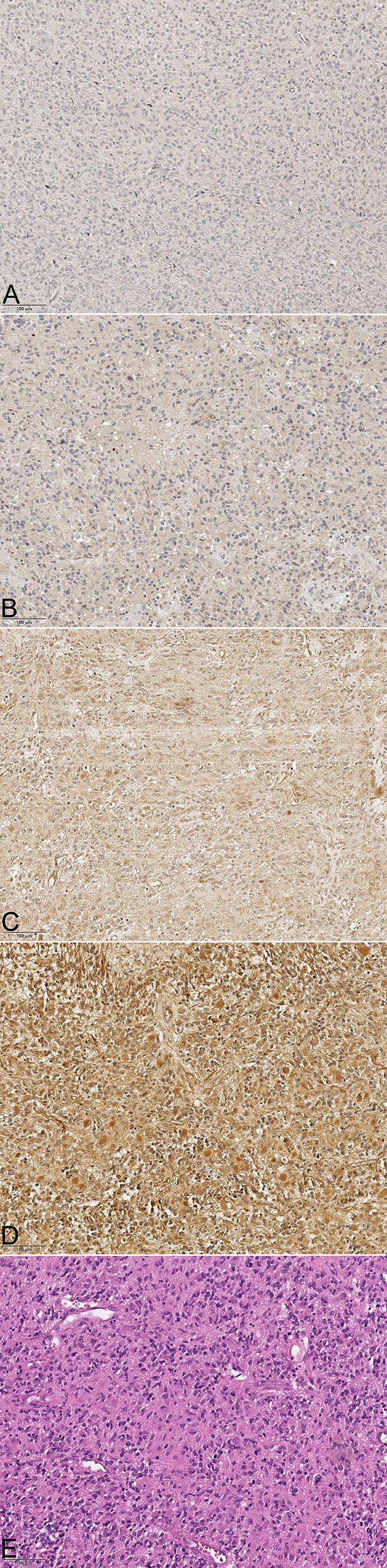
**a** Aromatase staining of GBM patients in each category (× 20). Picture **a** represents group 1 (0–10%), **b** is group 2 (11–40%), **c** group 3 (41–70%) and **d** is group 4 (71–100%). In picture **e** the hematoxylin eosin stainging is shown

Examples for each group of immunohistochemically stained tissue samples for ERα and aromatase as well as hematoxylin eosin are illustrated in Figs. 1 and 2.

The analysis of ERα staining demonstrated that 22 GBM tissue samples (12 female and 10 male) showed less than 10% immune positive tumor cells. Staining group 2 consisted of 20 tissue samples (8 female, 12 male) with 11–40% immune positive tumor cells. 13 tissue samples (5 female, 8 male) showed 41–70% of ERα immune positive tumor cells. 5 tissue samples demonstrated more than 70% of immunoreactive tumor cells, consisting of 4 female and 1 male patients.

The evaluation of the enzyme aromatase showed 2 tissue samples (2 female) with less than 10% of immune positive tumor cells. 8 GBM tissue samples, consisting of 4 female and 4 male patients, demonstrated 11–40% of immunoreactive cytoplasm. The staining group 3 consisted of 11 GBM tissue samples (8 female, 3 male) and 39 tissue samples showed more than 70% of immoreactive positive tumor cells (15 female, 24 male).

### GBM patient characteristics

The GBM cohort of the present study consisted of 60 patients (29 female, 31 male), with an age range from 25 to 85 years and an median age of 60 years for female and 58 years for male patients. The survival time of GBM patients after surgery ranged from 22 to 1920 days, with a median survival time for females of 411 days and male patients of 432 days. The patients received cross total resections followed by Stupp protocol consisting of radiation and chemotherapy. The patients had a Karnofsky index greater than 60%. MGMT status is known for 57 of 60 patients. 29 patients have no methylation of the MGMT and 28 patients present a methylation. There is no statistically significant correlation between the expression of ERalpha or Aromatase and the MGMT status.

### Survival analysis regarding ERα and Aromatase

**Fig. 3:**
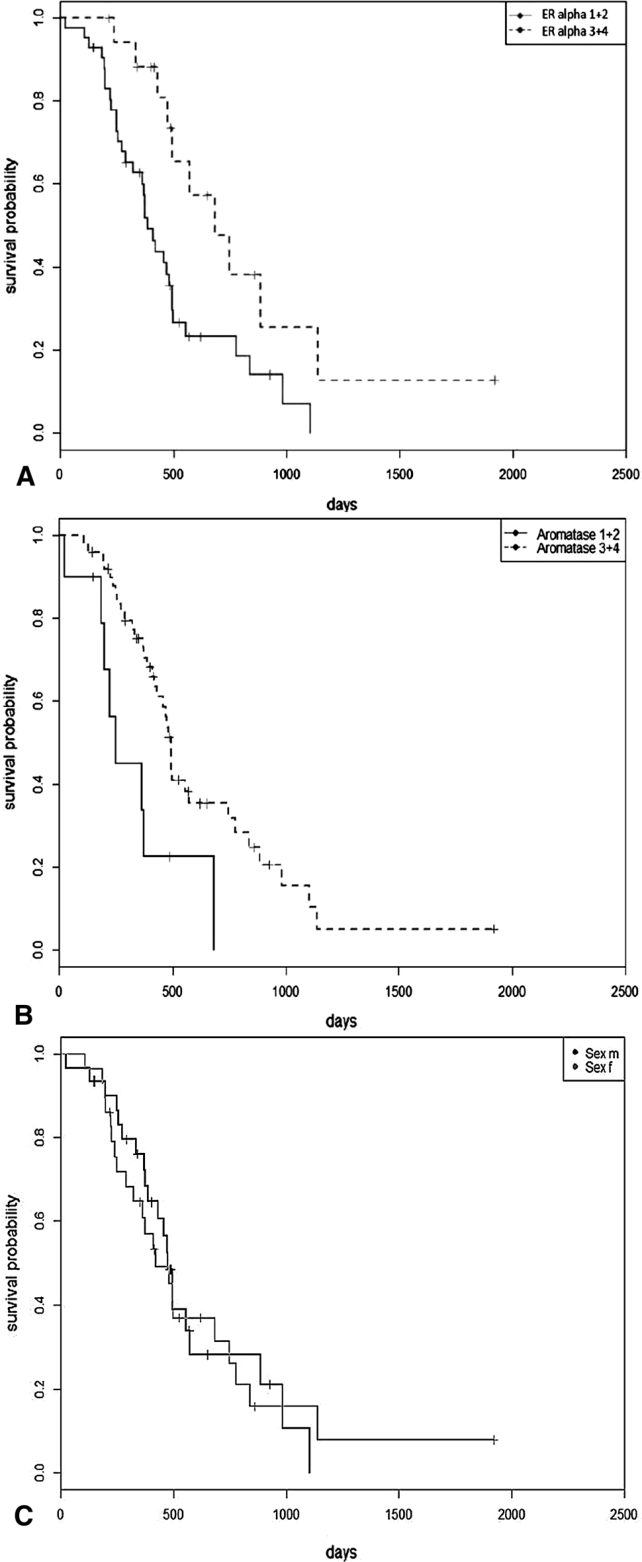
Kaplan–Meier survival curves for glioblastoma patients and the expression of ERα and aromatase. **a** High expression of ERα (dashed line, assessment group 3 and 4) was associated with significantly longer survival (p = 0.011), compared to low expression (continuous line, assessment group 1 and 2). **b** Significantly longer survival (p = 0.0104) for glioblastoma patients with high aromatase expression (dashed line, assessment group 3 and 4), compared to low enzyme expression. **c** Survival analysis for male (m) and female (f) GBM patients showed no difference between the groups (p > 0.89)

For survival analysis the staining intentions were judged as low expression with lower 40% of immune positive tumor cells (staining group 1 + 2) and high expression consisting of tissue samples with higher 40% of immune positive tumor cells (staining group 3 + 4). The Kaplan–Meier analysis showed that high ERα expression (> 40% immunoreactive tumor cells) in GBM patients was associated with significantly longer survival (Log rank: p = 0.0111, Fig. 3a), compared to low expression of ERα. Patients in the staining groups 3 + 4 showed a median survival of 492 days, whereas patients rated in staining groups 1 + 2 of ERα lived 371 days on median. GBM patients in high ERα staining groups lived 121 days on median longer than patients rated in staining group 1 + 2. The same effect was seen in GBM patients with high aromatase expression (> 40% immunoreactive tumor cells, Log rank: p = 0.0104, Fig. 3b), compared to low expression of the enzyme. GBM patients with low expression of aromatase lived 236 days on median, compared to high expression with a median survival time of 444 days, which was a difference of 208 days. No gender difference in the survival of female and male GBM patients appeared (Log rank: p = 0.89, Fig. 3c). The median survival time for female patients was 411 days and for male 432 days. Kaplan–Meier analyses were performed under adjusting to sex and BMI.

### Combined estradiol and TMZ therapy on GBM cell lines

**Fig. 4:**
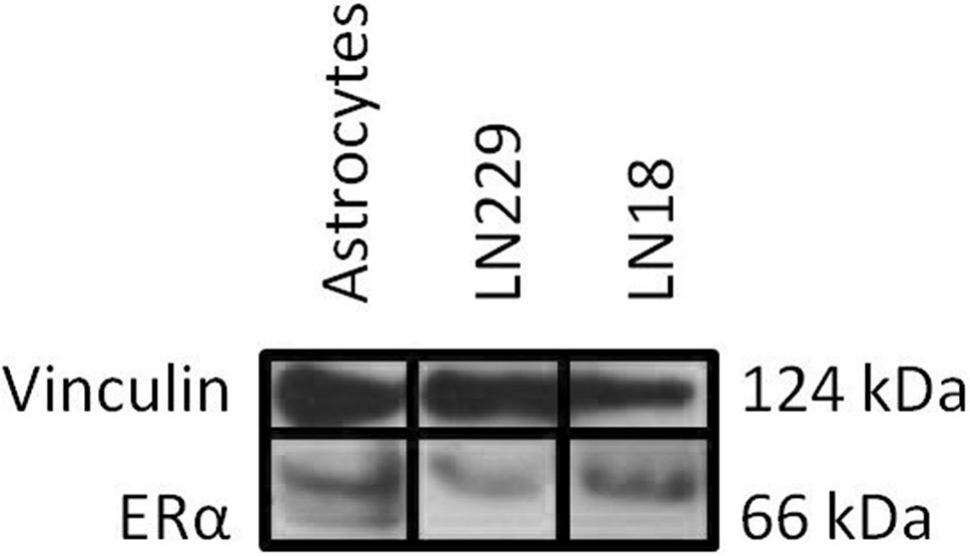
Western blot analysis of the cell lines LN229 and LN18. Vinculin was used as housekeeping gene. Both cell lines showed a distinct band for estrogen receptor alpha (ERα, 66 kDa, Western blot optical density for ERα: LN229: 1.16, LN18: 0.67). Primary astrocytes were used as positive control (Western blot optical density for ERα 0.74)

Before estradiol treatment a Western blot analysis was done, which confirmed that both cell lines LN18 and LN229 express ERα (Fig. 4).

Calculation of the half maximal inhibitory concentration (IC_50_) of estrogen and TMZ was performed after therapy with estradiol alone and after the combined estradiol and TMZ treatment.

**Fig. 5:**
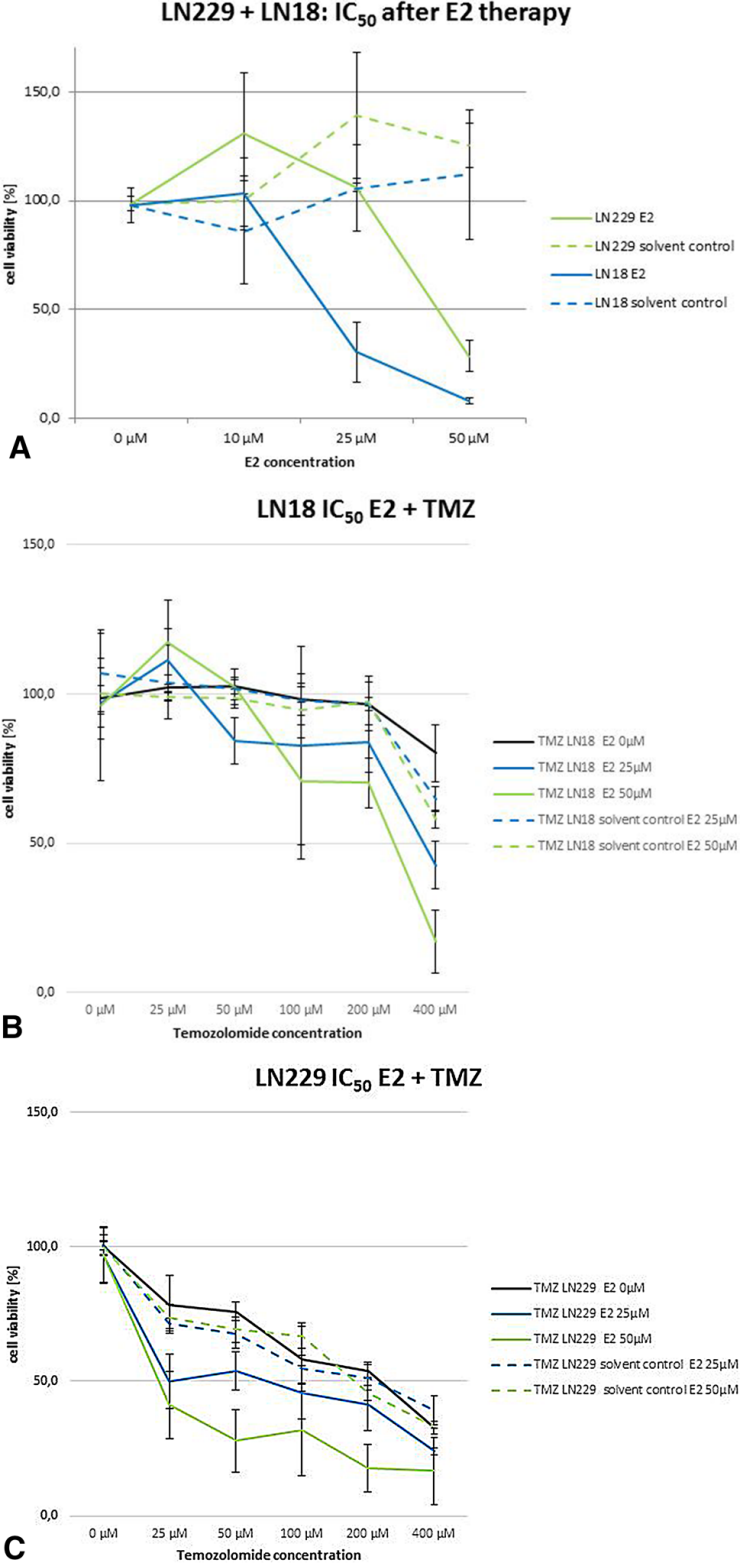
**a** IC_50_ calculation for LN229 (green) and LN18 (blue) after the therapy with 10 µM, 25 µM and 50 µM 17β-estradiol (E2). For LN229 cells a significantly lower cell viability was seen for 50 µM E2 (IC_50_ = 43.11 µM). Despite LN18 cells showed an IC_50_ of 20.96 µM E2. **b** IC_50_ calculation for LN18 after the therapy with 17β-estradiol (E2) and TMZ in different dosing regimens. The cells with the pre-treatment of 25 µM and 50 µM E2 showed a significantly lower cell viability compared to the control. The treatment with 10 µM E2 (not shown in the diagram) was equal to the control. **c** IC_50_ calculation for LN229 after the therapy with E2 and TMZ in different dosing regimens. The cells with the pre-treatment of 25 µM and 50 µM E2 showed a significantly lower cell viability compared to the control. The treatment with 10 µM E2 (not shown in the diagram) was equal to the control

High concentrations of estradiol resulted in significantly lower tumor cell viability, compared to control (Fig. 5a). The IC_50_ for the cell line LN229 was 43.11 µM 17β-estradiol (188.01 µM for solvent control) and for LN18: 20.96 µM 17β-estradiol (solvent control: − 191.14 µM). After 17β-estradiol and chemotherapy significantly lower cell viability, i.e. stronger sensitivity against TMZ, was seen for the estradiol concentrations 25 µM and 50 µM for both cell lines (Figs. 5b and 5c).

## Discussion

GBM still has an unfavorable prognosis, despite combined therapy. Interestingly, higher incidence rates can be observed for male patients. Moreover, animal studies have shown that GBM tumor growth is sex specific [11, 12]. The neurobiological reason for this gender difference remains unclear, and it seems obvious that sexual hormones, like estrogen, may play a key role in the development and tumor proliferation. Since estrogen binds to ERs it is possible that the amount of ERs could be a survival factor for men and women.

To further elucidate this hypothesis we analyzed the expression of sexual hormone receptor ERα and the hormone converting enzyme aromatase in male and female GBM patients, in relation to patients’ survival times. By immunohistochemistry we were able to show that high expression of ERα and aromatase was associated with longer patient survival, gender independently. On the basis of these results we investigated the direct effect of estradiol in combination to TMZ on glioblastoma tumor cells in vitro. We were able to show that estrogen treatment decreased tumor cell viability and increased the sensitivity to TMZ.

Our cohort consists of 60 GBM patients with a similar amount of men (31) and women (29). The median age of both groups is also similar (female: 60 years, male: 58 years), suggesting high comparability of the groups. All patients received a cross total resection followed by adjuvant radiation and chemotherapy. The patients had a Karnofsky index greater than 60%. Further information regarding individual drug intake was not known for our cohort.

Whereas previous studies commonly focused on ERs or hormone converting enzymes [24, 25], we performed immunohistochemistry for both: ERα and aromatase. Selective estrogen receptor inhibitors, as well as Selective Estrogen Receptor Modulators, inhibit the growth of gliomas and induce apoptosis [26–29], indicating that estrogen modulates the tumor growth via the classical nuclear estrogen receptors. The synthesis, regulation and also the effect of estradiol is not only influenced by glial tumor cells but also by the microenvironment, particularly by reactive astrocytes. These cells that surround the tumor margins are another source for estradiol and express ERs and aromatase [15]. Although we investigated the vital core region of the tumor and therefore evaluated the regions of highest tumor cell content, definite distinction between reactive astrocytes and tumor cells is not always possible morphologically. Since glial tumors have a truly diffusely infiltrating growth pattern within the brain, to date only IDH1/IDH2-mutated glioma cells can be sharply distinguished form reactive glia, using immunostaining. In this study, we examined only IDH1/IDH2-wildtype GBMs.

From epidemiological studies we know that the incidence of GBM is 1.6–2.0 times higher in men than in women [4]. This effect was especially pronounced between groups of pre-menopausal women compared to a male cohort. This gender advantage disappears in post-menopausal women [19]. However, our GBM patient cohort showed gender-independent survival times. One reason for this effect could be that the cohort was more homogeneous with mainly post-menopausal women (median age 60).

Jimenez et al. analysed different types of glial tumors and showed that estrogen concentrations were highest in GBM biopsies compared to low grade astrocytomas and were directly related to high aromatase expression [30]. In addition, they showed that the mRNA expression of ERα was higher than the expression of ERβ and the immunoreactivity of aromatase and estrogen receptors decreased with higher grades of tumor malignity. They also reported that patients with low ERα were associated with the worst prognosis, which is concordant with our findings. In their study mRNA levels of aromatase were inversely correlated to the survival time. In our study, we investigated aromatase protein levels in a homogeneous IDH1-wildetype GBM cohort, whereas Jimenez et al. analysed different glial tumors of different biological background and grades of malignity. In addition, it should be noted that measuring mRNA-levels in a tumor lysate is a different approach to studying immunohistochemical expression in individual cells within a tissue.

In breast cancer, high dose estrogen therapy (5–100 µM) on ER positive breast cancer cells leads to inhibition of tumor proliferation and increases the percentage of S-, G2- and M-phase, by decreasing the percentage of G0 and G1 cells [31]. In our study the glioma cell lines were treated with a high dosage of 17β-estradiol (10 µM, 25 µM, 50 µM) over 48 h as a pretreatment before chemotherapy. TMZ was given over 5 days, similar to one cycle in clinical treatment. The TMZ dosage was based on the calculated IC_50_ dosages for the cell lines LN18 and LN229 [32]. As expected, the pretreated cells showed a higher sensitivity against TMZ, compared to control.

Estrogen interacts with the nuclear ERα and β but the distinct functions of these receptor types are still unclear. ERα knockout mice suffered from more pronounced brain tissue damage after induced hypoxia compared to ERβ knockout mice that showed no difference to the untreated control group [20]. This supports our finding that ERα plays an important role in tumor suppression and represents an important survival factor.

As anticipated, the therapy with estradiol, in a dose depending manner, resulted in a slower tumor growth rate of LN18 and LN229 [24]. While low dose treatment (10 µM estradiol) was equal to the control, higher concentrations led to a decrease in cell viability.

To the authors knowledge this is the first experimental study of a combined 17β-estradiol and chemotherapy treatment on glioblastoma cell lines. Diverse studies with 2ME2 (metabolite of estrogen) therapy in combination to radiation [33], as well as selective ERβ agonist combined with chemotherapy [26, 34] or selective estrogen receptor modulators with TMZ and radiation [28], showed a sensitization against chemotherapy or radiation and a decrease in cell viability. After estradiol pre-treatment a sensitization against TMZ was seen. However, it is difficult to distinguish which ER subtype is responsible for this effect. Further studies are necessary to get a deeper insight in the molecular mechanisms of ER subtypes.

Despite the neuroprotective effects of estradiol in the brain, it leads to many unwanted side effects like feminization in men, increased risk of cardiovascular disease, thrombosis and different types of cancer, especially invasive breast cancer [35]. More knowledge will be essential to design special targeted therapy with lower risks for side effects.

## Conclusion

High expression of ERα and aromatase showed an advantage in GBM patients’ survival. In vitro therapy with estradiol demonstrated stemming effects on tumor progression. The administration of estradiol before conventional postoperative chemotherapy and radiation may be beneficial to glioblastoma patients and will have to be examined in further studies and clinical trials.
